# A computational study exploring echinoderm-derived compounds for inhibition of aminoglycoside acetyltransferases

**DOI:** 10.1371/journal.pone.0327409

**Published:** 2025-07-01

**Authors:** Abdullah R. Alanzi, Moneerah J. Alqahtani, Jawaher H. Alqahtani, Hattan A. Alharbi

**Affiliations:** Department of Pharmacognosy, College of Pharmacy, King Saud University, Riyadh, Saudi Arabia; Laurentian University, CANADA

## Abstract

Aminoglycoside acetyltransferases (AACs) catalyze the acetylation of aminoglycoside antibiotics, playing a major role in bacterial resistance and posing a serious threat to global healthcare. Despite growing interest in natural products, echinoderm-derived metabolites remain largely unexplored as AAC inhibitors. This study presents a comprehensive *in-silico* investigation into the potential of these marine compounds to inhibit four key AAC enzymes: Aminoglycoside 2′-N-acetyltransferase, AAC(3)-Ib, AAC(6′)-Im, and AAC(3)-Iva. A virtual screening of 1600 echinoderm metabolites was performed using molecular docking, ADMET profiling, and 100 ns molecular dynamics simulations. The top 10 compounds against each enzyme were shortlisted based on binding affinity, with CMNPD15515, CMNPD17440, CMNPD3088, and CMNPD29853 showing the most stable interactions and highest binding energies. These compounds consistently outperformed native aminoglycoside ligands in docking scores and MMGBSA binding free energy calculations, suggesting stronger inhibitory potential. While a few top hits exhibited violations of Lipinski’s Rule of Five particularly in molecular weight their strong target engagement and stable dynamic profiles support their candidacy as lead molecules. This work underscores the evolutionary and structural uniqueness of echinoderm metabolites as a promising reservoir for antibiotic adjuvants. It also establishes a computational framework for prioritizing marine natural products against antibiotic resistance targets. Although experimental validation remains essential, this study provides compelling early evidence to guide future in vitro and in vivo research toward the development of novel AAC inhibitors.

## 1. Introduction

An increasing number of gram-negative bacteria are swiftly developing resistance to the majority, and in some instances, all currently utilized antibiotics. This escalation in resistance poses significant challenges in treating severe infections caused by multidrug-resistant (MDR) bacteria, rendering it increasingly difficult and costly [1,2]. New treatments for resistant organisms demand innovative antibiotics and adjuvants that enhance the efficacy of existing drugs. This strategy aims to prolong the effectiveness of antibiotics that are currently in use but are losing efficacy due to the proliferation of resistance traits. Notably, this approach has yielded success for β-lactams, with numerous β-lactam/ β-lactamase inhibitor formulations currently in practice. However, other categories of antibiotic resistance inhibitors have yet to progress beyond the confines of research laboratories [3–5].

Aminoglycosides are bactericidal antibiotics that impair translational accuracy, resulting in the production of faulty proteins and ultimately causing cell death. They are particularly effective against serious gram-positive and gram-negative infections, especially when used in combination with other antimicrobial agents [6–9]. Enzymatic inactivation is the most frequent kind of resistance that bacteria have developed against aminoglycosides, even though they have several resistance mechanisms. There have been multiple reports of chemicals that interfere with antibiotic molecule inactivation via various molecular processes or promote cellular absorption [8,10–12]. None of them could be developed into therapeutic formulations, despite their demonstrated efficacy [13,14].

A variety of mechanisms are used by bacteria to develop resistance to aminoglycosides are decreased absorption of aminoglycosides, chemical modifications of aminoglycosides made possible by enzymes that modify aminoglycosides, and mutation or methylation of particular 16S rRNA nucleotides linked to aminoglycoside binding [15,16]. Among these strategies, the most prevalent and clinically significant is the enzymatic modification of aminoglycosides. Three classes of enzymes responsible for this modification have been identified: ATP-dependent nucleotidyltransferases (O-nucleotidylation, ANT), ATP-dependent phosphotransferases (O-phosphorylation, APH), and acetyl coenzyme A (CoA)-dependent N-acetyltransferases (N-acetylation, AAC). Four distinct types of AACs employ acetyl-CoA to acetylate the amino groups in aminoglycoside antibiotics: AAC (1), AAC (2′), AAC (3), and AAC (6′) [17–19]. AAC(2′) enzymes, exemplified by AAC(2′)-Ia found in Providencia stuartii, are known to enhance the acetylation of gentamicin, dibekacin, netilmicin, kanamycin, and tobramycin, with their expression heightened in the presence of aminoglycosides. AAC (2′) enzymes are a clinically important subclass of aminoglycoside acetyltransferases that confer resistance to aminoglycosides such as gentamicin, kanamycin, and tobramycin. These enzymes are chromosomally encoded in gram-negative bacteria such as *Providencia stuartii* and are upregulated in the presence of aminoglycosides, enhancing their resistance capability [18]. AAC (3)-I enzymes, including AAC(3)-Ia and AAC(3)-Ib, are prevalent among *Enterobacteriaceae* and non-fastidious gram-negative nonfermenters, providing resistance to sisomicin, gentamicin, and fortimicin (astromicin) through minor mutations within each lineage [20–22]. The AAC (6′) enzymes represent the most numerous groups of AACs, with over 40 characterized members found in both gram-negative and gram-positive bacteria. These enzymes are divided into two more categories: The distinct aminoglycoside inactivation patterns of AAC (6′)-I and AAC (6′)-II allow for their differentiation. AAC (6′)-I enzymes, except for gentamicin C1, normally provide resistance against a range of aminoglycosides, such as amikacin and gentamicin C1a and C2. In contrast, AAC (6′)-II enzymes catalyze the acetylation of all types of gentamicin but not amikacin [7,14,23].

AACs hinder antibiotics from binding to bacterial targets, making it more difficult to kill bacteria, and reducing their effectiveness. Understanding AAC types and characteristics is crucial for developing resistance-countering measures [11]. The purpose of this study is to utilize *in-silico* approaches to identify such inhibitors of aminoglycoside antibiotics that can act as inhibitors causing disruption in the normal metabolic pathways of these antibiotics. This research addresses the existing gap by utilizing metabolites derived from echinoderms to target aminoglycoside-modifying enzymes, specifically Aminoglycoside 2′-N-acetyltransferase, AAC (3)-Ib, AAC (6′)-Im, and AAC (3)-Iva. Echinoderms which include sea cucumbers, sea urchins, and sea stars—are known to produce a wide array of structurally diverse secondary metabolites, such as triterpene glycosides, steroids, peptides, and polysaccharides. These bioactive compounds possess various pharmacological properties, including antimicrobial, anticancer, anti-inflammatory, and antiviral activities. Notably, the distinct chemical frameworks of echinoderm metabolites, which differ significantly from those of terrestrial sources, present promising new scaffolds for drug discovery. Recent studies have highlighted the potent antimicrobial properties of lipid extracts from echinoderms against various pathogens, including antibiotic-resistant strains. The bioactive compounds in these extracts, such as polyunsaturated fatty acids (PUFAs), disrupt bacterial membranes and interfere with essential cellular processes, leading to bacterial cell death. In light of the growing challenge of antibiotic resistance, investigating metabolites from echinoderms offers a valuable strategy for identifying novel antimicrobial compounds. [24]. These compounds often disrupt bacterial membranes or inhibit essential enzymes, suggesting potential efficacy against AAC enzymes. The distinctive structural characteristics of echinoderm metabolites including sulfated groups and amphipathic nature may enhance their ability to interact with bacterial enzymes such as AACs. Consequently, our emphasis on compounds derived from echinoderms is driven by their evolutionary adaptations and proven antimicrobial activity, which underscore their potential as effective AAC inhibitors. [25].

While the unique chemical scaffolds of echinoderm-derived metabolites offer promising leads for AAC inhibition, their natural availability and routes to synthesis also merit careful consideration. Echinoderm metabolites are typically present at low levels often micro-grams to milligrams per organism resulting in limited extraction yields; for example, sea cucumbers of the family Cucumariidae collectively yielded only 68 triterpene glycosides across nine species in one survey, highlighting scarcity in nature [26,27]. To overcome supply bottlenecks, advanced extraction techniques such as supercritical CO₂, enzyme-assisted, and pressurized solvent methods have been applied successfully to marine invertebrates [27], and sustainable approaches including mariculture and heterologous expression of echinoderm biosynthetic gene clusters in microbial hosts have demonstrated scalable production of marine natural products [28,29]. From a synthetic chemistry perspective, the dense stereochemistry and sp³-rich frameworks of polyhydroxynaphthazarins and triterpene glycosides pose challenges; nevertheless, total syntheses of dimeric (poly)hydroxynaphthazarins and chemo-enzymatic routes to echinoderm gangliosides have been reported, illustrating viable synthetic pathways [30,31]. Moreover, AI-driven retrosynthetic planning platforms such as Spaya enable rapid generation of optimized synthetic routes from commercially available precursors, thereby accelerating analogue design and lead optimization [32]. These combined strategies of sustainable sourcing, biotechnological production, and modern synthetic methodologies ensure that echinoderm-derived compounds can serve as practical and innovative leads in the development of aminoglycoside acetyltransferase inhibitors.

Previous research on aminoglycoside acetyltransferase (AAC) inhibition has largely focused on synthetic and microbial-derived compounds, many of which demonstrated preliminary in vitro or *in-silico* efficacy but failed to transition into viable therapeutic leads due to pharmacokinetic or mechanistic limitations. For example, a study by Garneau-Tsodikova et al. (2016) reported the identification of barbituric acid derivatives that showed competitive inhibition of AAC (6′)-Ib; however, these compounds did not significantly restore aminoglycoside efficacy in resistant bacterial strains, possibly due to limited membrane permeability or metabolic instability [33,34]. Similarly, pyrrolidine pentamine analogs reported by Magaña et al. (2023) showed promising in vitro IC₅₀ values in the micromolar range but exhibited steep structure-activity relationship constraints and required structural rigidification to enhance stability and cellular activity [35]. Moreover, while metal complexes such as zinc and copper pyrithione have been explored for their inhibitory effects on AAC (6′)-Ib, their broad-spectrum toxicity and lack of selectivity limit their clinical utility [36]. These challenges highlight the urgent demand for innovative molecular scaffolds that not only exhibit high binding affinity and suitable pharmacokinetic profiles but also possess the potential for efficient synthetic optimization.

By integrating molecular docking, ADMET profiling, and molecular dynamics simulations, this study not only identifies novel inhibitors but also establishes a robust framework for their systematic evaluation. The primary novelty lies in its focus on echinoderm metabolites, an underexplored frontier in AAC inhibition. Unlike prior studies that predominantly investigate terrestrial or microbial natural products, this research delves into the vast yet untapped marine biodiversity of echinoderms. By targeting unique chemical scaffolds with the potential to counteract aminoglycoside resistance, the findings promise to enhance the therapeutic utility of aminoglycosides and contribute significantly to combating antibiotic resistance on a global scale.

## 2. Methodology

### 2.1. Preparation of ligands

Metabolites derived from echinoderms were retrieved from the Comprehensive Marine Natural Products Database (https://www.cmnpd.org/). The phytochemical structures were then prepared using the LigPrep module in Maestro [37]. The geometry of each ligand was optimized, leading to the production of 32 conformers. Energy minimization of the compounds was performed using the empirical OPLS_2005 force field [38]. The ligands were saved in the “.mae” format for use in the docking process.

### 2.2. Structure validation & molecular docking studies

Prior to docking, 3D structures of all proteins were validated through PROCHECK present at Saves server v6.0 (https://saves.mbi.ucla.edu/) to ensure the structures are ready to dock with no clashes and perfect residue score. This study aims to perform virtual screening of echinoderm-derived metabolites against a range of aminoglycoside-modifying enzymes. The selected target proteins for this investigation include Aminoglycoside 2′-N-acetyltransferase (PDB ID: 1M44), AAC (3)-Ib (PDB ID: 4YFJ), AAC (6′)-Im (PDB ID: 6BFF), and AAC (3)-Iva (PDB ID: 7MQL). Prior to molecular docking, the protein structures were refined and optimized using the Protein Preparation Wizard [39]. During the receptor preparation process, multiple steps were carried out, such as forming disulfide bonds, assigning zero-order bonds to metal ions, and adding hydrogen atoms. Extra ligands and crystallographic water molecules were eliminated. For optimization, the pKa values of ionizable groups were refined at pH 7.0 using the PROPKA software [40]. Energy minimization was carried out using the OPLS_2005 force field. Following protein preparation, three-dimensional grids were generated at the predicted active sites of each protein to facilitate site-specific docking. The compounds were docked using the Standard Precision (SP) mode of the Glide module from the Schrödinger Suite. This module employs an empirical scoring function known as Glide Score, which estimates the binding free energy of ligands by evaluating factors such as van der Waals forces, electrostatic interactions, hydrogen bonding, hydrophobic enclosure, desolvation effects, and other contributing elements to rank the docking poses [41]. Additionally, the ADMET characteristics and physicochemical properties of the docked compounds were evaluated using the QikProp tool [42].

### 2.3. Molecular dynamics simulation

The complexes were evaluated for protein conformation and ligand stability through a 100 ns simulation conducted using Desmond [43]. The objective of this study is to conduct virtual screening of metabolites derived from echinoderms against a selection of aminoglycoside-modifying enzymes. The target proteins chosen for this analysis include Aminoglycoside 2′-N-acetyltransferase (PDB ID: 1M44), AAC (3)-Ib (PDB ID: 4YFJ), AAC (6′)-Im (PDB ID: 6BFF), and AAC (3)-Iva (PDB ID: 7MQL). Before docking procedures, these protein structures were processed and optimized using the Protein Preparation Wizard [44]. To mimic physiological conditions, counter ions were added to neutralize the system, along with 0.15 M NaCl salt. The simulations were run under the NPT ensemble at a temperature of 300 K and a pressure of 1 ATM. Before starting the production run, the systems were relaxed, and trajectories were recorded every 50 ps using a time step of 2 fs. The trajectories were then analyzed using the simulation interaction diagram module of Desmond.

## 3. Results

### 3.1. Structure’s validation

**Fig 1:**
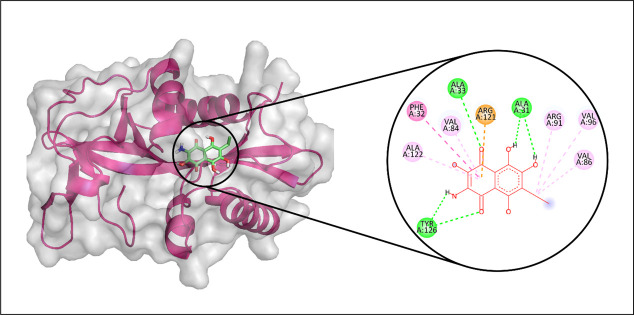
Ramachandran plots from PROCHECK for PDB IDs 1M44, 4YFJ, 6BFF, and 7MQL. Each structure exhibits >90% of residues in the most favored region, with only 6BFF and 7MQL showing minor (<1%) outliers (marked in red), localized to peripheral, flexible loop regions rather than the core or active site.

All four PDB structures (1M44, 4YFJ, 6BFF, 7MQL) were analyzed using PROCHECK, revealing that each scored above 90% of residues in the “most favored” regions well within standards for high-quality crystallographic models (Fig 1). Structures 6BFF and 7MQL, while of high resolution (1.7 Å and 1.6 Å, respectively) and low refinement statistics (R_work ≤ 0.18, R_free ≈ 0.20), displayed a handful (<1%) of residues in disallowed regions. Importantly, these outliers are exclusively located in surface-exposed loop segments with inherently higher flexibility and partial electron density support, as confirmed by visual inspection. Such occurrences are neither unexpected nor problematic at this resolution, where PROCHECK may flag minor deviations despite correct modeling. Given their negligible proportion and non-critical positioning, these rare outliers do not compromise the overall structural integrity or validity of these models for docking studies, consistent with wwPDB validation guidelines.

### 3.2. Molecular docking and ADMET studies

The metabolites from echinoderms were docked against various AAC enzymes using the Glide tool’s standard precision protocol. Docking results were evaluated based on the Glide score, and the top 10 compounds for each receptor were selected (Table 1). Four control compounds Streptomycin, Apramycin, Ribostamycin, and Tobramycin were also docked with the receptors. In the Aminoglycoside 2′-N-acetyltransferase docking analysis, the controls exhibited binding affinities ranging from −5.471 to −4.764 kcal/mol, while the selected compounds showed affinities between −7.554 and −6.806 kcal/mol. For Aminoglycoside acetyltransferase AAC (3)-Ib, the controls’ docking scores ranged from −4.661 to −3.02 kcal/mol, whereas the selected compounds scored between −8.667 and −7.143 kcal/mol. In the case of Aminoglycoside acetyltransferase AAC (6′)-Im, the hits demonstrated binding affinities from −10.152 to −7.884 kcal/mol, outperforming the controls. In a similar trend, the identified hit compounds demonstrated stronger binding affinities toward Aminoglycoside acetyltransferase AAC (3)-Iva when compared to the reference controls. Additionally, the selected natural metabolites were evaluated for their physico-chemical and ADMET characteristics. Although one or two compounds showed minor deviations from Lipinski’s rule of five, the remaining pharmacokinetic parameters were within acceptable limits. These included “QPlogHERG” values under −5, “QPlogPo/w” ranging from −2.0 to 6.5, “QPlogBB” between −3.0 and 1.2, “QPPCaco” (classified as poor below 25 and excellent above 500), and “QPlogKhsa” within the range of −1.5 to 1.5 (Table 2).

**Table 1:** The selected echinoderms metabolites and their docking scores against each enzyme.

Sr. No.	Compounds	Glide Score
**Aminoglycoside 2**′**-N-acetyltransferase**		
**1.**	CMNPD15515	−7.554
**2.**	CMNPD17447	−7.535
**3.**	CMNPD17448	−7.346
**4.**	CMNPD2509	−7.215
**5.**	CMNPD25649	−7.212
**6.**	CMNPD28335	−7.135
**7.**	CMNPD9368	−7.098
**8.**	CMNPD3097	−6.856
**9.**	CMNPD15554	−6.832
**10.**	CMNPD11602	−6.806
**11.**	*Streptomycin	−5.391
**12.**	*Apramycin	−4.764
**13.**	*Ribostamycin	−5.341
**14.**	*Tobramycin	−5.471
**Aminoglycoside acetyltransferase AAC (3)-Ib**		
**1.**	CMNPD17440	−8.667
**2.**	CMNPD16818	−7.921
**3.**	CMNPD15521	−7.918
**4.**	CMNPD15587	−7.734
**5.**	CMNPD16458	−7.718
**6.**	CMNPD15573	−7.626
**7.**	CMNPD16452	−7.446
**8.**	CMNPD4621	−7.325
**9.**	CMNPD10048	−7.179
**10.**	CMNPD15551	−7.143
**11.**	*Streptomycin	−4.661
**12.**	*Apramycin	−3.564
**13.**	*Ribostamycin	−3.781
**14.**	*Tobramycin	−3.02
**Aminoglycoside acetyltransferase AAC (6**′**)-Im**		
**1.**	CMNPD3088	−10.152
**2.**	CMNPD17447	−9.387
**3.**	CMNPD24290	−8.878
**4.**	CMNPD31462	−8.794
**5.**	CMNPD14618	−8.548
**6.**	CMNPD13149	−8.397
**7.**	CMNPD15608	−8.15
**8.**	CMNPD5280	−8.061
**9.**	CMNPD29842	−8.04
**10.**	CMNPD2142	−7.884
**11.**	*Streptomycin	−2.452
**12.**	*Apramycin	−5.228
**13.**	*Ribostamycin	−6.808
**14.**	*Tobramycin	−4.617
**Aminoglycoside acetyltransferase AAC(3)-Iva**		
**1.**	CMNPD29853	−9.259
**2.**	CMNPD15626	−8.979
**3.**	CMNPD2547	−8.401
**4.**	CMNPD23089	−8.326
**5.**	CMNPD13819	−8.311
**6.**	CMNPD2162	−8.263
**7.**	CMNPD7937	−8.2
**8.**	CMNPD2152	−8.191
**9.**	CMNPD10073	−8.185
**10.**	CMNPD13091	−8.117
**11.**	*Streptomycin	−6.066
**12.**	*Apramycin	−6.753
**13.**	*Ribostamycin	−6.542
**14.**	*Tobramycin	−6.309

* Control

**Table 2:** The ADMET profiles of the selected echinoderms metabolites predicted by QikProp.

Compounds	MW	HBD	HBA	QPlogPo/w	QPlogHERG	QPPCaco	QPlogBB	QPlogKhsa
**Aminoglycoside 2**′**-N-acetyltransferase**								
CMNPD15515	265.221	5	7	−0.553	−3.532	17.644	−2.24	−0.6
CMNPD17447	848.134	5	11	2.893	−6.436	18.588	−3.304	−0.157
CMNPD17448	296.238	3	7	−0.476	−4.776	30.129	−1.972	−0.598
CMNPD2509	742.901	6	13	2.016	−3.046	3.4	−3.77	−0.546
CMNPD25649	1237.39	14	27	−3.732	−6.302	0.641	−7.287	−3.099
CMNPD28335	253.166	6	8	−1.793	−3.294	4.146	−2.685	−0.801
CMNPD9368	728.917	8	13	0.921	−5.357	23.571	−3.613	−0.65
CMNPD3097	744.873	8	14	0.376	−3.356	0.543	−4.846	−0.951
CMNPD15554	812.227	7	9	7.65	−6.033	41.27	−5.963	0.572
CMNPD11602	1155.424	14	21	0.152	−2.917	0.013	−11.262	−2.894
**Aminoglycoside acetyltransferase AAC(3)-Ib**								
CMNPD17440	770.146	7	9	6.461	−5.989	41.883	−5.657	0.187
CMNPD16818	810.211	7	9	7.651	−6.626	15.027	−7.062	0.706
CMNPD15521	796.228	6	8	9.182	−6.387	113.623	−5.43	1.166
CMNPD15587	870.263	9	11	5.732	−5.938	4.973	−7.37	−0.137
CMNPD16458	706.015	8	10	3.574	−5.313	24.813	−5.43	−0.735
CMNPD15573	844.269	8	10	6.889	−6.196	17.986	−6.912	0.176
CMNPD16452	718.07	7	9	5.626	−5.568	95.458	−4.758	−0.099
CMNPD4621	730.89	7	13	0.758	−3.745	0.418	−5.277	−0.84
CMNPD10048	844.269	8	10	7.189	−6.424	8.039	−7.456	0.396
CMNPD15551	798.2	7	9	6.845	−5.53	35.086	−5.734	0.344
**Aminoglycoside acetyltransferase AAC (6**′**)-Im**								
CMNPD3088	514.56	7	10	−1.037	−3.756	0.016	−3.098	−0.6
CMNPD17447	848.134	5	11	3.145	−6.31	40.143	−2.767	−0.147
CMNPD24290	827.783	12	22	−5.79	−6.416	0.473	−6.605	−3.098
CMNPD31462	356.33	3	7	1.805	−4.923	44.978	−2.175	0.042
CMNPD14618	268.229	4	8	−1.733	−3.511	28.682	−1.898	−0.835
CMNPD13149	444.349	3	9	0.594	−1.227	0.156	−3.646	−0.889
CMNPD15608	582.732	5	9	2.766	−2.666	3.81	−3.164	0.036
CMNPD5280	863.005	8	17	0.201	−3.895	0.461	−5.586	−1.313
CMNPD29842	360.275	2	8	1.105	−3.053	6.282	−2.305	−0.522
CMNPD2142	400.486	4	10	−1.582	−1.928	0.081	−1.73	−0.857
**Aminoglycoside acetyltransferase AAC(3)-Iva**								
CMNPD29853	1265.357	13	28	−3.024	−4.96	0.011	−9.334	−2.845
CMNPD15626	1281.4	13	28	−2.629	−5.094	0.015	−9.391	−2.847
CMNPD2547	1187.29	9	25	−1.567	−4.056	0.081	−6.704	−2.12
CMNPD23089	1251.374	12	27	−2.791	−5.239	0.011	−9.852	−2.907
CMNPD13819	938.291	11	15	2.813	−5.706	2.775	−8.031	−1.456
CMNPD2162	1429.514	16	33	−4.91	−5.629	0.002	−12.179	−4.086
CMNPD7937	837.632	5	10	−2.34	−3.627	0.345	−3.465	−1.374
CMNPD2152	1413.515	15	32	−3.893	−4.8	0.012	−9.431	−3.556
CMNPD10073	1413.515	16	32	−4.961	−4.87	0.002	−10.511	−3.807
CMNPD13091	816.215	8	10	6.432	−6.024	20.906	−6.314	0.079

### 3.3. Molecular interaction and simulation analysis

Molecular docking analyses were carried out to evaluate the interactions between echinoderm-derived metabolites and the selected protein targets: Aminoglycoside 2′-N-acetyltransferase, AAC (3)-Ib, AAC (6′)-Im, and AAC (3)-Iva. These evaluations were performed using Discovery Studio. Based on the docking scores obtained from Glide, the compounds CMNPD15515, CMNPD17440, CMNPD3088, and CMNPD29853 emerged as top candidates and were subsequently chosen for molecular dynamics simulations against Aminoglycoside 2′-N-acetyltransferase, AAC (3)-Ib, AAC (6′)-Im, and AAC (3)-Iva, respectively.

#### 3.3.1. Aminoglycoside 2′-N-acetyltransferase.

**Fig 2:**
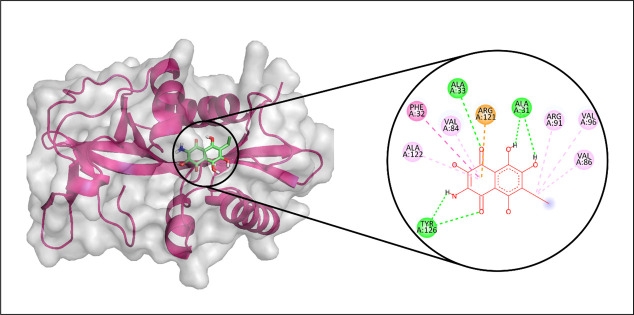
The molecular interaction of CMNPD15515 against Aminoglycoside 2′-N-acetyltransferase; (Left) Purple color shows the protein structure in helices and loops while light grey is the surface with transparency to some extent and green colored smaller structure is CMNPD15515 docked in binding site, prepared by PyMol. (Right) Enlarged 2D interactions of CMNPD15515 against Aminoglycoside 2′-N-acetyltransferase; Hydrogen bonds (green), Hydrophobic (magenta); Pi-Anion (orange).

**Fig 3:**
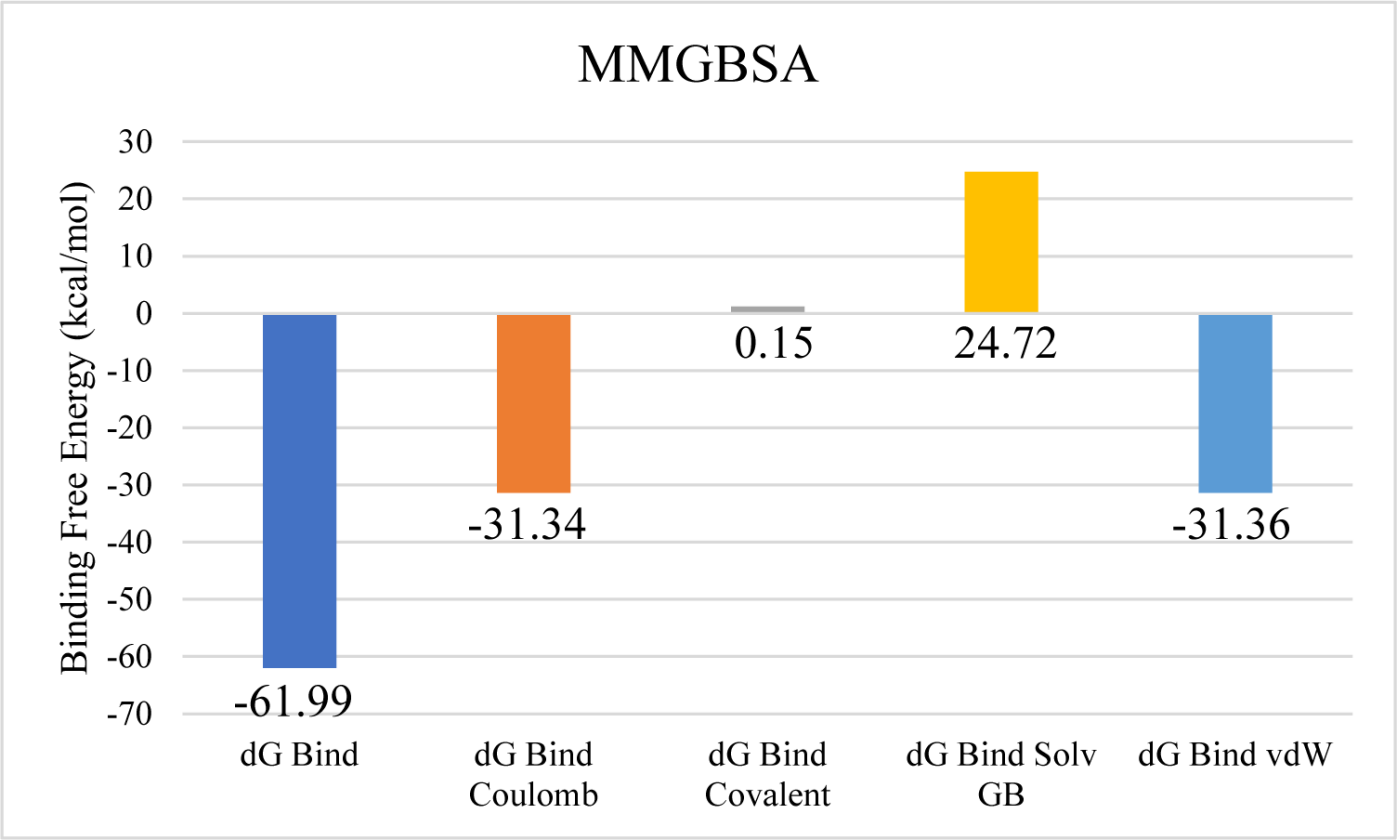
(A) The RMSD plot of the Aminoglycoside 2′-N-acetyltransferase complex depicting the root mean square deviation of protein c-alpha atoms (blue line) and ligand (red line) throughout the simulation time. **(B)** The residual fluctuation analysis of protein’s backbone; y-axis shows the RMSF values while x-axis shows the residues throughout the simulation time. **(C)** The protein-ligand interactions fraction shows individual residues taking part in some kind of bonding with the ligand where y-axis shows interaction fraction and x-axis shows the residue 3 letter code with its number; H-bonds (green), Hydrophobic (magenta), Ionic (pink), and Water Bridges (blue). **(D)** Percentage of interactions observed in snapshots.

**Fig 4:**
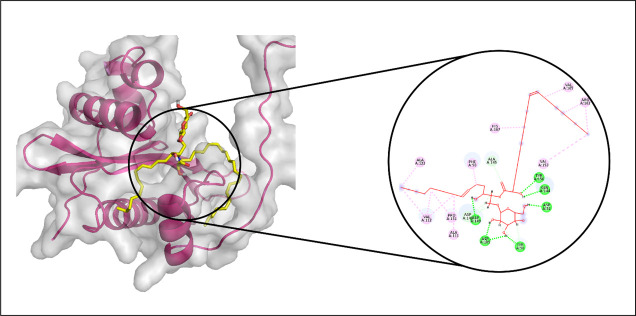
The binding free energy of the aminoglycoside 2′-N-acetyltransferase complex and the contribution of its energy components where y-axis shows the energy values in kilo calory per mol and x-axis shows the different energy components with bars depicting different energy levels.

Among the metabolites screened against Aminoglycoside 2′-N-acetyltransferase, CMNPD15515 demonstrated the strongest binding affinity. Molecular interaction analysis indicated that it established three hydrogen bonds with residues ALA33, ALA31, and TYR126. Furthermore, it formed seven hydrophobic contacts involving VAL86, VAL96, ARG91, ARG121, VAL84, PHE32, and ALA122, as depicted in Fig 2. To evaluate conformational stability and structural fluctuations of the complex, a 100 ns molecular dynamics simulation was performed, and the root mean square deviation (RMSD) of the Cα atoms was subsequently analyzed [45,46]. Throughout the simulation, the RMSD of the protein′s Cα atoms remained within a stable range of approximately 1.5 to 2.5 Å until around 80 ns, after which it temporarily rose to 4 Å before stabilizing back to its initial range near 90 ns. The RMSD trajectory of the ligand mirrored that of the protein, suggesting the overall stability of the protein-ligand complex during the simulation (Fig 3A). Additionally, root mean square fluctuation (RMSF) analysis was performed to assess the flexibility of individual protein residues in response to ligand binding [47]. RMSF analysis revealed that the majority of the protein residues experienced limited fluctuations during the molecular dynamics simulation, with values remaining under 2 Å indicating that ligand binding had minimal impact on the protein’s overall structural integrity. In contrast, higher flexibility was observed in certain loop regions, where fluctuations peaked at approximately 3.5 Å (Fig 3B). The protein-ligand complex remained stabilized through a network of interactions, including hydrogen bonds, hydrophobic contacts, ionic interactions, and water bridges. Notably, residues such as ALA31, ALA33, VAL84, VAL86, SER117, SER119, and TYR126 were actively involved in hydrogen bonding (Fig 3C), with Ala33 displaying the highest interaction frequency, present in 33% of simulation frames (Fig 3D). To gain deeper insights into complex stability, binding free energy was estimated using the Prime MM-GBSA approach. The total binding free energy was calculated as −61.99 kcal/mol, composed of contributions from Van der Waals (−31.36 kcal/mol), Coulombic (−31.34 kcal/mol), solvation (24.72 kcal/mol), and covalent (0.15 kcal/mol) energies (Fig 4).

#### 3.3.2. Aminoglycoside acetyltransferase AAC (3)-Ib.

**Fig 5:**
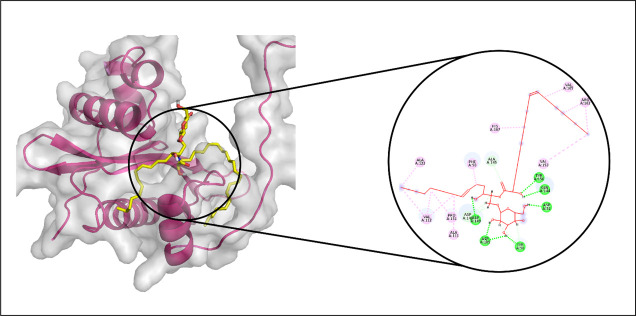
The molecular interactions of CMNPD17440 against Aminoglycoside acetyltransferase AAC (3)-Ib. (Left) Purple color shows the protein structure in helices and loops while light grey is the surface with transparency to some extent and yellow colored smaller structure is CMNPD17440 docked in binding site, prepared by PyMol. (Right) Enlarged 2D interactions of CMNPD17440 against Aminoglycoside 2′-N-acetyltransferase; Hydrogen bonds (green), Hydrophobic (magenta).

**Fig 6:**
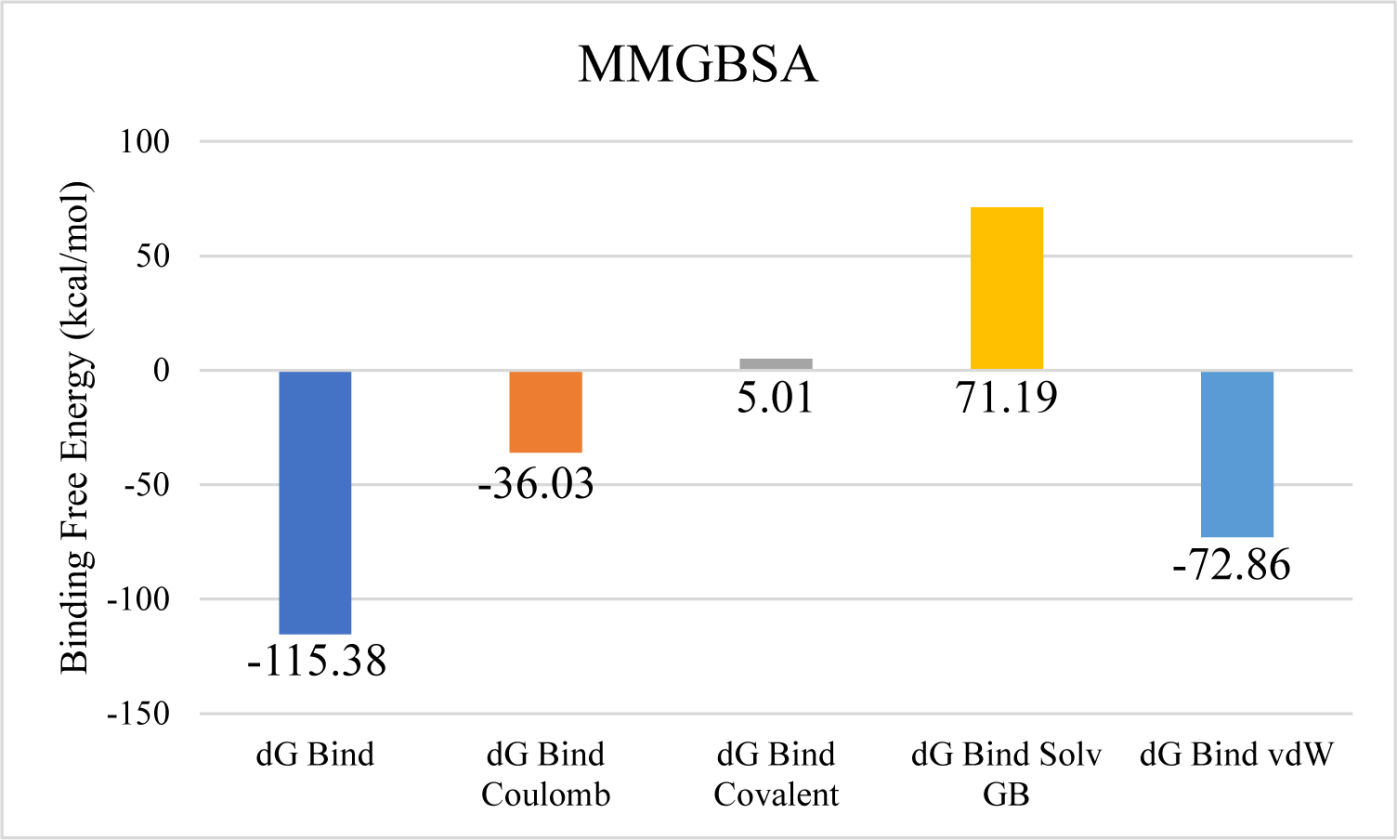
(A) The RMSD plot of the Aminoglycoside acetyltransferase AAC (3)-Ib complex with CMNPD17440 depicting the root mean square deviation of protein c-alpha atoms (blue line) and ligand (red line) throughout the simulation time. **(B)** The residual fluctuation analysis of protein’s backbone; y-axis shows the RMSF values while x-axis shows the residues throughout the simulation time. **(C)** The protein-ligand interactions fraction shows individual residues taking part in some kind of bonding with the ligand where y-axis shows interaction fraction and x-axis shows the residue 3 letter code with its number; H-bonds (green), Hydrophobic (magenta), Ionic (pink), and Water Bridges (blue). **(D)** Percentage of interactions observed in snapshots with a 2D interaction, and black line around the ligand.

**Fig 7:**
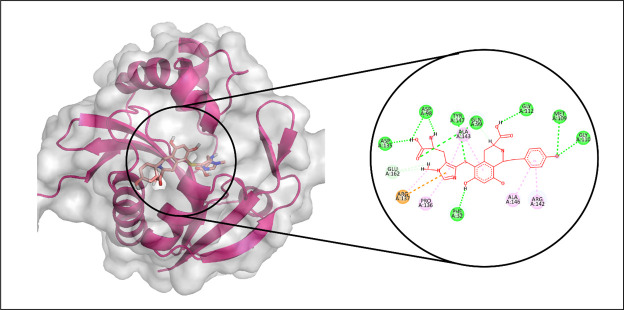
The overall binding free energy in the aminoglycoside acetyltransferase AAC (3)-Ib complex and the contribution of its energy components where y-axis shows the energy values in kilo calory per mol and x-axis shows the different energy components with bars depicting different energy levels.

The docking analysis of Aminoglycoside acetyltransferase AAC (3)-Ib identified CMNPD17440 as the ligand with the highest binding affinity, making it the candidate for subsequent molecular interaction and stability assessments. Molecular interaction studies revealed that CMNPD17440 formed hydrogen bonds with six residues: Tyr156, Gln144, Asp52, Tyr56, Asp109, and Asp149. Additionally, it engaged in nine hydrophobic interactions, as illustrated in Fig 5. RMSD analysis of the protein’s Cα atoms indicated structural stability, with values stabilizing between approximately 4.2 Å and 4.8 Å after equilibration occurred around 15 ns. The ligand’s RMSD profile closely paralleled that of the protein, suggesting sustained stability of the complex throughout the simulation period (Fig 6A). RMSF analysis further demonstrated that most protein residues remained compact and structurally consistent, except for two loop regions that displayed elevated flexibility, with fluctuations reaching approximately 4.8 Å and 3.2 Å, respectively (Fig 6B). Analysis of protein-ligand contacts revealed the presence of hydrogen bonds involving ASP52, ASP109, LEU110, GLN144, ASP149, and TYR156. Among these, ASP52 and ASP109 maintained frequent hydrogen bonding, while Asp149 also engaged in ionic interactions (Fig 6C). ASP149 emerged as the most frequently interacting residue, present in 71% of the simulation frames (Fig 6D). Binding free energy calculations, performed using the Prime MM-GBSA approach, showed a total binding free energy of −115.38 kcal/mol. Additional energy component details are presented in Fig 7.

#### 3.3.3. Aminoglycoside acetyltransferase AAC (6′)-Im.

**Fig 8:**
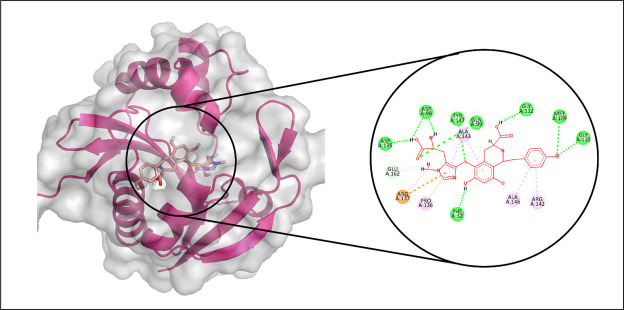
The molecular interactions of CMNPD3088 against Aminoglycoside acetyltransferase AAC ( [6]′**)-Im.** (Left) Purple color shows the protein structure in helices and loops while light grey is the surface with transparency to some extent and orange colored smaller structure is CMNPD3088 docked in binding site, prepared by PyMol. (Right) Enlarged 2D interactions of CMNPD3088 against Aminoglycoside 2′-N-acetyltransferase; Hydrogen bonds (green), Hydrophobic (magenta); Pi-Anion (orange).

**Fig 9:**
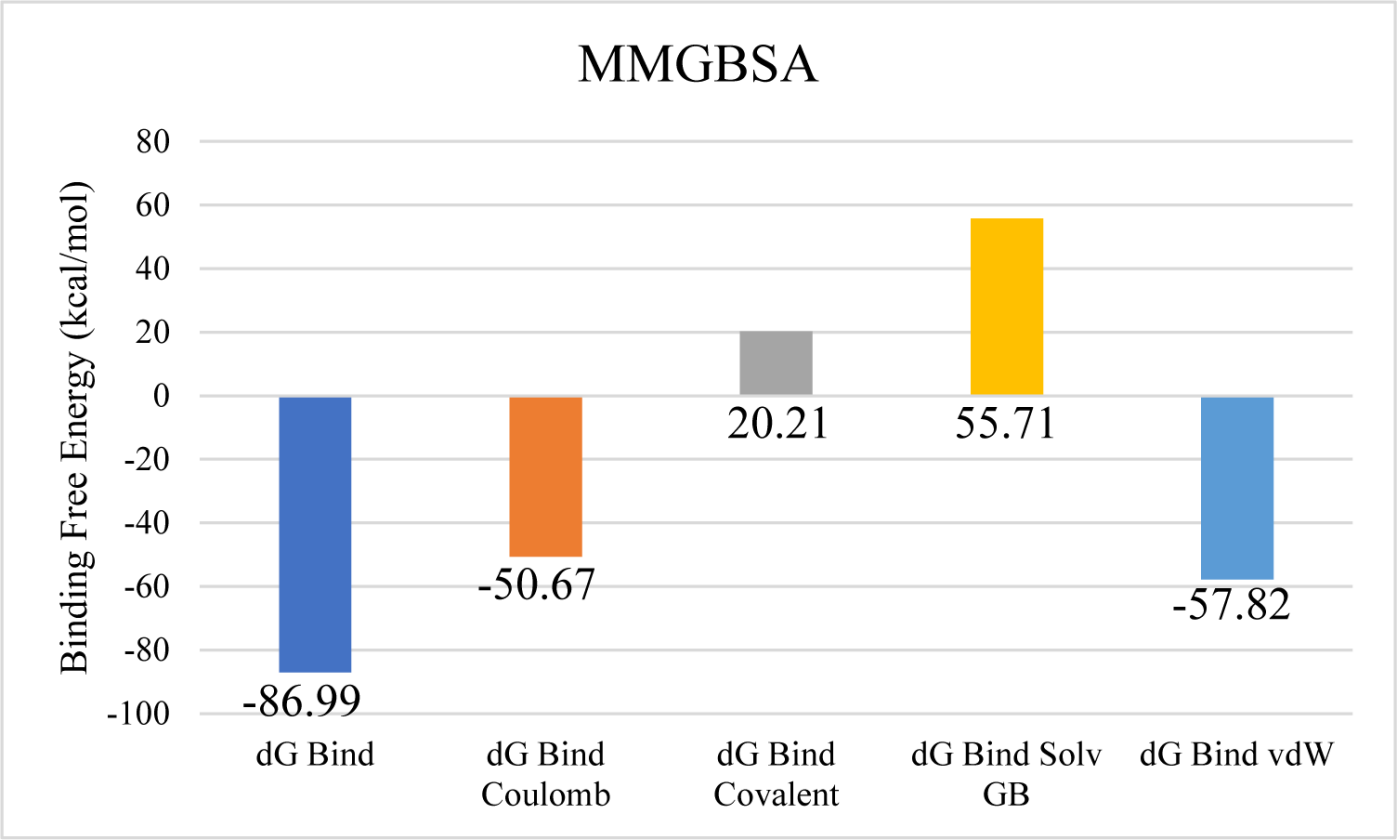
(A) The RMSD plot of the Aminoglycoside acetyltransferase AAC (6′)-Im complex depicting the root mean square deviation of protein c-alpha atoms (blue line) and ligand (red line) throughout the simulation time. **(B)** The residual fluctuation analysis of protein’s backbone; y-axis shows the RMSF values while x-axis shows the residues throughout the simulation time. **(C)** The protein-ligand interactions fraction shows individual residues taking part in some kind of bonding with the ligand where y-axis shows interaction fraction and x-axis shows the residue 3 letter code with its residue number; H-bonds (green), Hydrophobic (magenta), Ionic (pink), and Water Bridges (blue). **(D)** Percentage of interactions observed in snapshots with a 2D interaction, and black line around the ligand.

**Fig 10:**
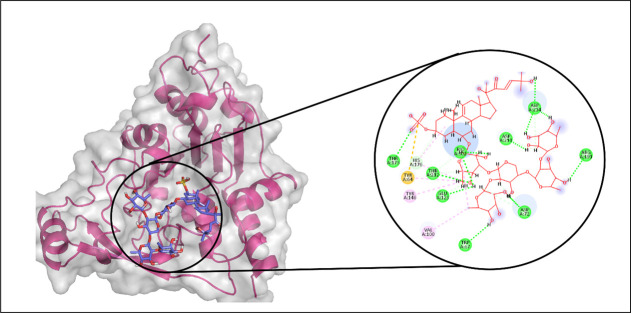
The overall binding free energy of the aminoglycoside acetyltransferase AAC ( [6]′**)-Im complex and the contribution of its energy components where y-axis shows the energy values in kilo calory per mol and x-axis shows the different energy components with bars depicting different energy level.**

Among the tested compounds, CMNPD3088 demonstrated the strongest binding affinity toward the aminoglycoside acetyltransferase AAC (6′)-Im. Molecular interaction analysis showed that this compound formed eight hydrogen bonds with key residues, including ASP135, ASP98, TYR147, GLN99, MET109, GLY110, GLY112, and PHE32. In addition, it established four hydrophobic interactions with PRO136, ALA143, ALA146, and ARG142, as well as a pi-sulfur interaction involving ARG137 (Fig 8). The RMSD profile of the protein’s Cα atoms gradually increased to around 2.4 Å by 30 ns, followed by a decline to 1.5 Å at 60 ns, where it remained stable for the remainder of the simulation. The ligand’s RMSD exhibited a similar pattern, indicating a stable complex formation (Fig 9A). RMSF analysis confirmed structural rigidity across most residues, with a notable exception in a loop region where fluctuations reached approximately 2.5 Å (Fig 9B). Further analysis of protein-ligand contacts identified multiple hydrogen-bonding residues, including PHE32, TYR33, ASP98, ILE101, TYR106, MET109, GLY110, ILE111, GLY112, ARG137, ASN139, and TYR147. Additionally, ionic interactions were detected with ALA113, Asp135, ARG142, LYS149, and GLU162 (Fig 9C). Among these, TYR147 exhibited the most frequent interactions, appearing in 89% of the simulation frames (Fig 9D). The total binding free energy, calculated using MM-GBSA, was −86.99 kcal/mol, with detailed energy contributions presented in Fig 10.

#### 3.3.4. Aminoglycoside acetyltransferase AAC (3)-IVa.

**Fig 11:**
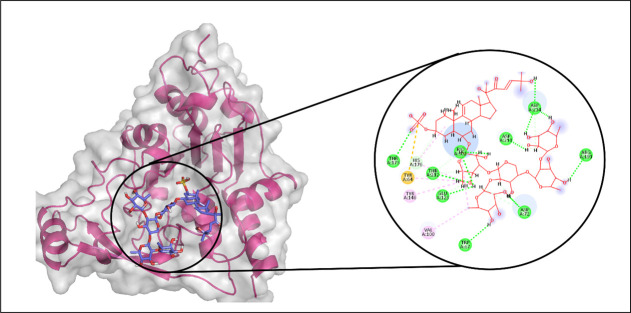
The molecular interactions of CMNPD29853 against Aminoglycoside acetyltransferase AAC [3]-IVa. (Left) Purple color shows the protein structure in helices and loops while light grey is the surface with transparency to some extent and blue colored smaller structure is CMNPD29853 docked in binding site, prepared by PyMol. (Right) Enlarged 2D interactions of CMNPD29853 against Aminoglycoside 2′-N-acetyltransferase; Hydrogen bonds (green), Hydrophobic (magenta); Pi-Anion (orange).

**Fig 12:**
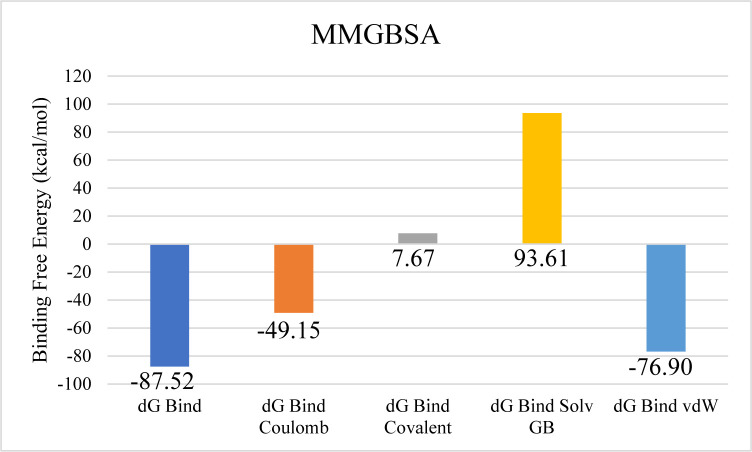
(A) The RMSD plot of the Aminoglycoside acetyltransferase AAC [3]-IVa complex depicting the root mean square deviation of protein c-alpha atoms (blue line) and ligand (red line) throughout the simulation time. **(B)** The residual fluctuation analysis of protein’s backbone; y-axis shows the RMSF values while x-axis shows the residues throughout the simulation time. **(C)** The protein-ligand interactions fraction shows individual residues taking part in some kind of bonding with the ligand where y-axis shows interaction fraction and x-axis shows the residue 3 letter code with its residue number; H-bonds (green), Hydrophobic (magenta), Ionic (pink), and Water Bridges (blue). **(D)** Percentage of interactions observed in snapshots with a 2D interaction, and black line around the ligand.

In the docking analysis targeting Aminoglycoside acetyltransferase AAC (3)-IVa, CMNPD29853 emerged as the top-performing ligand based on binding affinity. Molecular interaction analysis indicated that CMNPD29853 established eight hydrogen bonds with residues THR173, THR212, GLU123, TRP67, ASP72, ARG191, ASP214, and ASP211. Additionally, hydrophobic interactions were observed with VAL100 and TYR146, as illustrated in Fig 11. The RMSD trajectory of the protein’s C-alpha atoms remained stable, fluctuating between approximately 1.8 and 2.4 Å during the simulation period. The RMSD of the ligand mirrored that of the protein, signifying the formation of a stable complex (Fig 12A). RMSF analysis revealed that most residues exhibited minimal movement, except for flexible loop regions, which showed deviations of up to ~4.5 Å (Fig 12B).

**Fig 13:**
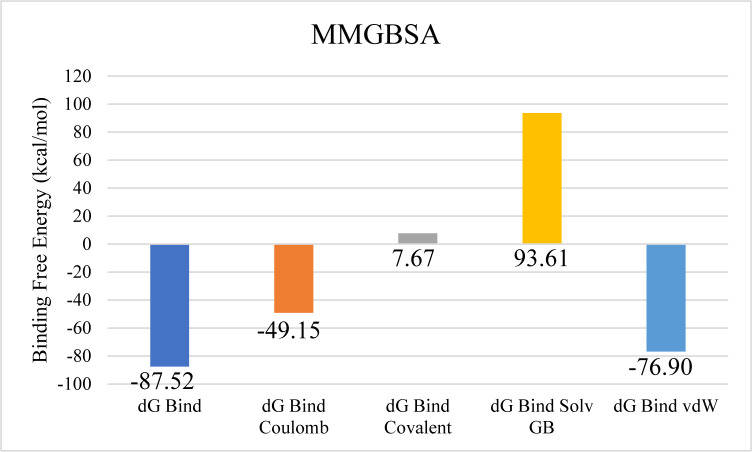
The overall binding free energy of the aminoglycoside acetyltransferase AAC (3)-Iva complex and the contribution of its energy components and the contribution of its energy components where y-axis shows the energy values in kilo calory per mol and x-axis shows the different energy components with bars depicting different energy level.

Further examination of protein-ligand contacts identified multiple residues contributing to hydrogen bonding, including PRO71, ASP72, ASP75, ASP79, LYS82, GLU123, TYR146, THR173, ArG191, THR212, and TYR219. GLU123 was also found to be involved in ionic interactions (Fig 12C). Among these, Glu123 demonstrated the highest interaction frequency, appearing in 76% of the simulation trajectory frames (Fig 12D). The total binding free energy, estimated via the Prime MM-GBSA approach, was −87.52 kcal/mol, with the detailed energy component breakdown shown in Fig 13.

## 4. Discussion

Aminoglycoside antibiotics are a class of drugs used to treat bacterial infections by inhibiting bacterial protein synthesis. Due to their wide therapeutic range and effective bactericidal action, aminoglycoside antibiotics are useful drugs for treating bacterial infections. They do, however, have drawbacks like toxicity and resistance. Aminoglycoside inhibitors are needed to address these problems by improving the overall effectiveness of these antibiotics, lowering their toxicity, and thwarting resistance processes [11,48]. This study addresses this critical need by introducing echinoderm metabolites as novel inhibitors, leveraging cutting-edge computational techniques to identify potential therapeutic candidates.

The integration of computer-aided drug design (CADD) methodologies in this study underscores the robustness of our approach. By combining molecular docking, ADMET profiling, and molecular dynamics (MD) simulations, we systematically identified and validated promising inhibitors. Unlike traditional drug discovery methods, CADD allows for rapid screening and optimization of compounds, reducing time, cost, and labor while maintaining high precision. Our results demonstrate that echinoderm metabolites possess unique chemical scaffolds and significant bioactivity, distinguishing this work from existing studies on AAC inhibition. Understanding how ligands bind, interact with, and inhibit specific proteins may aid in the identification of therapeutic options for a given disease [49–51].

The goal of this study is to screen echinoderm metabolites that can inhibit aminoglycoside antibiotics. The Comprehensive Marine Natural Products Database was used to obtain a library of echinoderm metabolites encompassing 1600 chemicals. The proteins used in this study were Aminoglycoside 2′-N-acetyltransferase, Aminoglycoside acetyltransferase AAC (3)-Ib, Aminoglycoside acetyltransferase AAC (6′)-Im, and Aminoglycoside acetyltransferase AAC (3)-Iva. We selected Aminoglycoside 2′-N-acetyltransferase (PDB ID: 1M44) for docking due to its established role in modifying aminoglycosides at the 2′-amino group, which is crucial for their antibacterial activity [52]. Furthermore, the availability of a high-resolution crystal structure makes this enzyme a suitable and reliable target for structure-based virtual screening.

Targeting AAC (2′) represents a promising approach to restore the effectiveness of aminoglycoside antibiotics by inhibiting their enzymatic inactivation. A library of echinoderm-derived metabolites was screened against multiple AAC enzymes using the standard precision mode of the Glide docking tool. The resulting docking scores (Glide GScore) were used to select the top 10 compounds for each receptor. Additionally, four known aminoglycosides—Streptomycin, Apramycin, Ribostamycin, and Tobramycin were included as reference compounds.

In the docking analysis with Aminoglycoside 2′-N-acetyltransferase, the control compounds exhibited binding affinities between −5.471 and −4.764 kcal/mol, whereas the selected echinoderm metabolites showed stronger binding, ranging from −7.554 to −6.806 kcal/mol. For Aminoglycoside acetyltransferase AAC (3)-Ib, control docking scores ranged from −4.661 to −3.02, while the selected metabolites ranged from −8.667 to −7.143 kcal/mol. Similarly, the top hits against AAC (6′)-Im demonstrated superior binding affinities, between −10.152 and −7.884 kcal/mol, outperforming the controls. A comparable trend was observed for AAC (3)-Iva, where the selected compounds also showed stronger binding than the reference ligands. To validate the reliability of the docking protocol, the native aminoglycoside ligands Streptomycin, Apramycin, Ribostamycin, and Tobramycin were re-docked into their respective AAC enzyme binding sites. These compounds served as positive controls and provided a benchmark for comparison with the test ligands. The GlideScores obtained for these native ligands were consistently lower (i.e., weaker binding) than those of several echinoderm-derived metabolites, confirming the capability of our docking setup to differentiate between weaker and stronger binders. This comparative analysis supports the reliability of the docking results and suggests that the selected marine metabolites may possess superior inhibitory potential.

ADMET profiling was performed to further support the pharmacokinetic suitability of the selected metabolites. Enhancing pharmacokinetic characteristics is essential in the drug development process to ensure that compounds can successfully progress through standard clinical trials and qualify as viable therapeutic candidates. In the present study, all identified compounds exhibited acceptable pharmacokinetic properties, and further assessments were subsequently conducted [53]. Although ADMET profiling indicated that some of the selected echinoderm-derived metabolites had low QPPCaco values (<25), suggesting limited intestinal absorption, this does not eliminate them as promising lead compounds. Molecules such as CMNPD15515, CMNPD17447, and CMNPD3088 showed strong binding affinities, stable interactions with target proteins, and favorable MMGBSA binding free energy values during molecular dynamics simulations. These properties suggest effective target engagement and potential pharmacological activity. The low predicted permeability is likely due to the structural complexity or size of natural products, challenges that can be mitigated through medicinal chemistry modifications, prodrug design, or alternative administration methods [54,55]. Such refinement is common in drug development pipelines, particularly for compounds derived from marine organisms, which frequently offer novel scaffolds that are underrepresented in conventional libraries [52]. Hence, these metabolites should be considered as preliminary scaffolds for further optimization rather than as fully developed drug candidates.

The compounds exhibiting the strongest binding affinities to their respective target proteins were selected for detailed interaction analysis. Among them, CMNPD15515 demonstrated the highest affinity for Aminoglycoside 2′-N-acetyltransferase; CMNPD17440 for Aminoglycoside acetyltransferase AAC (3)-Ib; CMNPD3088 for Aminoglycoside acetyltransferase AAC (6′)-Im; and CMNPD29853 for Aminoglycoside acetyltransferase AAC (3)-Iva.

In addition, molecular dynamics (MD) simulations provided crucial information regarding the conformational stability and dynamic characteristics of the protein–ligand complexes, reinforcing the reliability of the initial docking results. RMSD analysis and hydrogen bond evaluations confirmed that the complexes maintained structural stability throughout the simulation. Furthermore, MM-GBSA calculations indicated energetically favorable interactions, highlighting the therapeutic potential of the identified candidate inhibitors. These computational approaches not only refine the predictive power of molecular docking but also support ligand prioritization, guide structural optimization, and offer deeper insights into the molecular basis of protein–ligand interactions [56]. The findings from both MMGBSA and MD simulations suggested that the selected compounds remained stably bound within the active sites, indicating their potential as strong inhibitors [57,58]. To find inhibitors against aminoglycoside antibiotics, the study’s novel therapeutic targets may prove to be highly beneficial for the field of drug therapy. The implications of this work extend beyond AAC inhibition. This study contributes to the search for effective therapeutic options against aminoglycoside resistance by identifying novel scaffolds that demonstrate strong binding affinities and favorable pharmacokinetic properties. Although the computational analyses offer important initial evidence supporting the potential of echinoderm-derived metabolites as inhibitors of aminoglycoside acetyltransferases, it is important to recognize the intrinsic limitations associated with *in-silico* approaches. Molecular docking simulations offer predictions on binding affinities and interactions but may not fully account for the dynamic nature of proteins, the influence of solvent environments, or the complexity of cellular systems. Similarly, ADMET predictions are based on established models that may not capture all variables affecting a compound’s pharmacokinetics and toxicity [59,60].

In contrast to previous studies discussed and mentioned in the introduction section, our current study introduces a fundamentally different approach by harnessing the chemical diversity of echinoderm-derived metabolites an under-exploited class of marine natural products. By leveraging a library of 1600 structurally diverse echinoderm metabolites and applying a robust computational pipeline integrating molecular docking, ADMET profiling, and molecular dynamics simulations, we successfully identified compounds (e.g., CMNPD15515, CMNPD17440, CMNPD3088, and CMNPD29853) with GlideScores and MMGBSA binding energies significantly superior to those of native ligands such as Streptomycin and Tobramycin. Notably, CMNPD3088 exhibited a docking score of −10.152 kcal/mol against AAC (6′)-Im, far outperforming the native controls (e.g., Streptomycin, −2.452 kcal/mol), and maintained high stability throughout a 100 ns simulation with consistent RMSD values. Importantly, unlike many earlier studies that focus solely on either computational screening or enzyme kinetics, our integrated pipeline provides a holistic evaluation framework from structural preparation to dynamic interaction analysis offering a more predictive and translational insight into inhibitor performance. Thus, our study not only fills a critical gap in AAC-targeted drug discovery by presenting marine-derived inhibitors but also establishes echinoderm metabolites as viable and superior candidates for future preclinical development. This positions our work as a significant advancement in both methodology and therapeutic potential within the domain of antimicrobial resistance.

The computational pipeline employed in this study integrates multiple, well-established *in-silico* techniques namely, structure-based virtual screening, ADMET profiling, molecular dynamics (MD) simulations, and MM/GBSA binding free energy calculations to predict potential inhibitors of aminoglycoside acetyltransferases (AACs). This multifaceted approach enhances the reliability of our predictions by cross-validating results through different computational lenses. Notably, similar computational-only strategies have successfully identified bioactive compounds in prior studies. For instance, an *in silico*-based study successfully identified some lead compound against Cisplatin-Induced Renal Injury using molecular docking, *in-silico* predictions of the lead compounds and successfully predicted experimental outcomes using solely on *in-silico*-based prediction using MTT assays, animal models or rats, and rtPCR [61]. Not only this one study, but there are tens of thousands such studies like this, such as a recent study first utilized bioinformatics pipeline comprising different techniques to experimentally validate the *ILK* gene against prostate cancer using shRNA transfection, qPCR, and western blotting [62]. Another study similar to ours, using *in silico* analyses such as molecular docking, ADMET profiling as the sole base for their experimental validation including inoculum preparations, periodical dilutions successfully validated the antibacterial activity of pyrrolo[2,3-d],pyrimidine compounds especially compound RP-3 declaring it as a therapeutic agent in the fight against microbial infections [63]. These examples underscore the potential of comprehensive computational methodologies to not only predict but also guide the discovery of effective inhibitors, thereby justifying the standalone robustness of our *in-silico* approach.

While our study provides a comprehensive computational analysis of potential AAC inhibitors, we acknowledge the absence of experimental validation as a limitation. To address this, we have outlined a detailed plan for future experimental work. This includes the cloning and over-expression of AAC(3)-Ib and AAC (6′)-Im enzymes, followed by in vitro inhibition assays to determine the IC₅₀ values of the top candidate compounds identified in our study. Subsequent steps involve conducting minimum inhibitory concentration (MIC) assays using resistant bacterial strains to assess the efficacy of these compounds in a biological context. Furthermore, we plan to perform cytotoxicity assays on mammalian cell lines to evaluate the safety profile of the lead compounds. These experimental validations will not only substantiate our computational findings but also provide critical insights into the therapeutic potential and safety of the identified inhibitors, thereby bridging the gap between *in-silico* predictions and clinical applicability.

## Conclusion

This study highlights echinoderm metabolites as novel inhibitors of aminoglycoside acetyltransferases (AACs), addressing a critical gap in combating antibiotic resistance. By employing robust computational approaches, including molecular docking, ADMET profiling, and molecular dynamics simulations, promising candidates such as CMNPD15515, CMNPD17440, CMNPD3088, and CMNPD29853 were identified. The novelty of utilizing under-explored marine metabolites underscores the potential of this research to reinvigorate aminoglycoside efficacy. While further experimental validation is necessary to confirm the therapeutic potential of these compounds, this study provides a solid foundation for developing innovative treatments against AAC-mediated resistance and advancing global antibiotic resistance efforts.
